# Metabolic syndrome and body shape predict differences in health parameters in farm working women

**DOI:** 10.1186/s12889-018-5378-9

**Published:** 2018-04-04

**Authors:** Ilze Mentoor, Maritza Kruger, Theo Nell

**Affiliations:** Department of Physiological Sciences, Faculty of Natural Sciences, Stellenbosch University, Western Cape Stellenbosch, 7600 Republic of South Africa

**Keywords:** Metabolic syndrome, Body shape, Body composition

## Abstract

**Background:**

Sufficient evidence associate body shape to detrimental lifestyle diseases including the metabolic syndrome (MetS). The prevalence of the MetS, as well as effects of the MetS and body shape on body composition, insulin-like growth factor-1 (IGF-1), C-reactive protein (CRP) and sex hormone parameters were investigated in a female farm worker population in the Western Cape.

**Methods:**

Women between the ages of 20–60 years were classified according to the International Diabetes Federation’s definition of the MetS. Assessments included body shape (android/gynoid), blood pressure, anthropometric, bioelectrical impedance analyses and blood analyses for fasting glucose and insulin, lipid profile, IGF-1, CRP, and sex hormone parameters.

**Results:**

The prevalence of the MetS was 52%, with abdominal obesity 68.8%, hypertension 66.4% and low high density lipoprotein-cholesterol (HDL-c) levels (64.1%) being the more prevalent MetS risk factors. The MetS, irrespective of body shape, was found to be associated with body mass index (*p* < 0.01), fat mass (%) (*p* < 0.01), waist circumference (*p* < 0.001), HDL-c (*p* < 0.001), systolic blood pressure (*p* < 0.05) and diastolic blood pressure (*p* < 0.01). No significant differences were observed for IGF-1, CRP and sex hormone parameters.

**Conclusion:**

The prevalence of the MetS and its individual risk factors were found to be significantly high in this female farm worker population. Additionally, the study showed that the MetS, body shape and/or both could predict differences in body composition, physiological and biochemical parameters in women.

## Background

Following, and adapting to a westernized lifestyle (unhealthy eating habits, dietary changes and decreased physical activity) has significantly increased in both developed and developing countries globally [1]. This in turn has contributed to an increase in the incidence of obesity and various lifestyle related-diseases [2].

The metabolic syndrome (MetS), regarded as a major risk factor for chronic diseases of lifestyle, consists of a cluster of metabolic, physiological and biochemical risk factors, independently associated with cardiovascular disease, and diabetes mellitus [3]. The main constituents of the MetS include; abdominal obesity, dyslipidaemia, increased arterial blood pressure (systolic and diastolic), insulin resistance (IR) and impaired blood glucose homeostasis, dependent on the MetS definition used [3, 4]. However, due to the complexity of the MetS, there is still no unifying definition, which clearly defines the MetS and its diagnostic criteria [4].

The MetS has been extensively studied in various populations with evidence pointing toward a high prevalence globally, including South Africa [5–9]. There also appears to be sufficient evidence indicating that women present with a higher risk compared to men [7, 8]. More specifically, there is an increase in the prevalence of android body shape observed amongst women, exacerbating the global prevalence of the MetS and its individual components [10].

Body composition, i.e. increased body mass (BM), obesity and android body shape; have been associated with an increased risk for developing metabolic-related diseases, especially in women [10, 11]. An android body shape (apple shape), refers to adipose tissue present in the abdominal compartment, and is characterized with an increase in visceral adipose tissue (VAT), which is normally associated with a worsened metabolic risk factor profile (IR, hypertension, dyslipidaemia and inflammation). The gynoid body shape (pear shape) is mostly associated with adipose tissue distributed around the gluteo-femoral region of the body; however, its relationship with disease risk is still debateable [12, 13].

Generally, adipose tissue seems to be involved in the pathophysiology of the MetS, where it plays a role in sex hormone production, as well as providing a source of low-grade inflammation and hence involved in the development of IR [13, 14]. The MetS is characterised by a deregulated adipokine profile (increased interleukin-6 and C-reactive protein (CRP)), which in turn leads to chronic low-grade inflammation [14, 15]. Since adipokines has such diverse functions, a dysregulation in the synthesis of these adipokines and their actions in relation to obesity, have been linked to the MetS [16]. Furthermore, the MetS is also characterised by excessive androgen (decreased sex hormone binding globulin (SHBG) and increased testosterone) synthesis via the increased conversion of oestrogens and androgenic precursors in adipose tissue [17]. It has furthermore been hypothesised that the MetS is also associated with increased insulin-like growth factor-1 (IGF-1) synthesis, which can exacerbate the development of the individual MetS risk components [18]. The role of the MetS as a single entity and/or its components in inflammation, sex hormones and growth factors however still needs to be elucidated, since no single component can explain the complexity of the MetS pathophysiology.

Despite this, no MetS prevalence data exist for women in a farm working environment as well as the individual components in the Western Cape region of South Africa. Therefore, determining the prevalence of the MetS in this specific population as well as region of South Africa, will partly enable us to describe, understand and resolve associations of the MetS, and provide significant insights into the extent of the MetS in the Western Cape. The primary aim was to first describe the prevalence of the MetS in this female farm worker population, and then to classify these women based on their body shape, body composition, as well as selected biochemical parameters.

## Methods

### Study design, ethical considerations and recruitment

Ethical approval was obtained from the Human Research Ethics Committee of Stellenbosch University (protocol number N13/04/052). A cross-sectional, baseline descriptive study design was followed between March until July 2015.

Farming communities were identified, after which farm workers from three different wine estates including, Villiera at the Owethu Clinic (Stellenbosch), Neethlingshoff (Stellenbosch), and Solms-Delta Wine Estate (Franschhoek), were invited to attend information sessions regarding the specific research project. Farm workers could volunteer to participate, where after visitations were scheduled for each volunteer on different days during the week. At each scheduled visitation, the researcher verbally explained to the participant what was expected of them during the data collection process, and that there would be a time investment of approximately 30 min. Participants were given sufficient time to thoroughly read through the participant information leaflet and consent form, after which they were free to ask any questions. After reading through the informed consent form, written informed consent was obtained from all volunteering participants based on inclusion criteria. Participants were informed that they could withdraw at any time point, and were assured of their anonymity and confidentiality within the study.

### Body composition assessments

Body mass was measured using a Seca 634 automatic scale (Seca, United Kingdom, Birmingham, England) to the nearest 0.01 kg. Height was measured using a portable, standard stadiometer (Leicester™; Leicester, England), assuming the correct anatomical stance, to the nearest 0.1 cm. The body mass index (BMI) (kg/m^2^) was calculated using these base measurements. Waist circumference and hip circumference were assessed to the nearest 0.1 cm using a Lufkin tape measure (Lufkin, USA). Waist-to-hip ratio (WHR) was subsequently calculated using these measures.

Bio-electrical impedance analysis (BIA) measurements were performed using the multi-frequency Bioscan 920 II analyser (Maltron 920, UK), to assess fat mass (%) and muscle mass (kg).

Body shape was estimated using the WHR ratio as well as visual inspection of the volunteers standing in the anatomical position as being either android or gynoid. We excluded *n* = 7 due to inability to align calculated WHR with the visual presentation of the body shape.

### Study population

For this part of the study population, volunteering apparently healthy women between the ages of 20–60 years were included. Inclusion criteria included volunteers had to be, (i) women between the ages of 20–60 years old, (ii) residing in the Western Cape Province (Winelands region), (iii) all included women had to have all parameters for this part of the study measured, and (iv) must have been able to provide informed consent. Those who were excluded were either younger, or older than 20–60 years, not usual residents from the WestCape Province, pregnant or lactating at the time of data 132#?>collection, or excluded due to missing parameters as a result of technical issues.

### Selection of participants

The total number of successfully recruited volunteering participants (which initially included both men and women) were *n* = 191. From this sample, a sub-sample was drawn in which *n* = 63 were excluded; *n* = 42 men for this particular part of the study, *n* = 2 women who had withdrawn consent due to time constraints, *n* = 7 women due to the inability to classify their body shape, and *n* = 12 due to technical errors in data collection for the BIA measurements.

### Stratified sub-sampling and metabolic syndrome classification

Of the remaining *n* = 128 women, the group was subsequently classified using International Diabetes Federation (IDF) criteria as having the MetS. This definition includes the compulsory elevated waist circumference (WC) (≥ 80 cm for women), plus any two of the following components: (i) elevated blood pressure (systolic blood pressure (SBP) ≥ 130 mmHg, and diastolic blood pressure (DBP) ≥ 85 mmHg), (ii) elevated fasting blood glucose (FBG) (≥ 5.6 mmol/L), (iii) low high density lipoprotein-cholesterol (HDL-c) (< 1.3 mmol/L for women) or elevated triglycerides (TG) (≥ 1.7 mmol/L) [19]. Applying this classification, a total of *n* = 66 women were classified as having the MetS, while *n* = 62 participants were classified into a non-MetS group.

Female participants within these two respective groups were further classified according to body shape (gynoid or android), to render the four respective groups: MetS with gynoid body shape (MetSG) (*n* = 29), MetS with android body shape (MetSA) (*n* = 37), non-MetS with gynoid body shape (NMetSG) (*n* = 50), and non-MetS with android body shape (NMetSA) (*n* = 12).

Due to logistical reasons and time constraints, only *n* = 80 could be included for data and laboratory analyses. This sub-sample was randomly selected from the total *n* = 128, however, for the NMetSA group all were included. The n = 80 women were randomly allocated to the specific subgroups as follows: MetSG (*n* = 23), MetSA (*n* = 23), NMetSG (*n* = 22) and NMetSA (*n* = 12).

### Blood pressure

Blood pressure was measured in duplicate on the right arm, using a calibrated aneroid sphygmomanometer (Erka Perfect Aneroid 48, Germany), and stethoscope (Littmann 3 M stethoscope, USA) with an appropriate sized cuff after a stabilising period of ten minutes in a sitting position.

### Blood sampling and analysis

Plasma blood glucose, serum insulin and a full blood lipid profile were assessed through the chemical PathCare laboratories (Stellenbosch) to assist in classify women as having the MetS or not. Female serum testosterone, sex hormone binding globulin (SHBG) and free androgen index (FAI) were also assessed. Highly sensitive enzyme linked immunosorbent assay (ELISA) kits were used to quantify CRP (CRP human simple set ELISA kit®, Abcam, UK) [20], and IGF-1 concentration (IGF-1 human ELISA kit®, Abcam, UK) [21]. Serum samples were diluted 25,000 times for CRP, and 20 times for IGF-1. The optical densities of all participant samples were measured using an EL800 universal microplate reader (Bio-tek Instruments, South Africa) at 450 nm, within 15 min after the stop solution was added.

### Statistical analysis

All data was analysed using Statistica Software version 12 (StatSoft, Inc., USA). Significance was accepted at *p* < 0.05. All normally distributed results are reported as means and 95% confidence intervals (CI), and not normally distributed results as medians and interquartile ranges (IQR). For normally distributed data, student t-tests were performed to determine the difference in variables between two groups. Factorial analysis of variance (ANOVA) with Bonferonni post hoc test was done to establish significance between the four respective groups. The Mann-Whitney U test was employed to determine the difference in variables between two groups, and a one-way Kruskal-Wallis ANOVA was performed to determine the difference in variables between all the study groups, for data that was not normally distributed.

## Results

### Prevalence

From the total sample (*n* = 128 women), *n* = 66 women were identified with the MetS (52%), while *n* = 62 did not present with the MetS (48%). For all the women in this study (*n* = 128), the most prevalent individual risk factor was a high WC (68.8%, *n* = 88), followed by elevated blood pressure (BP) (66.4%, *n* = 85), and low HDL-c levels (64.1%, *n* = 82), with approximately 26.6% presenting with elevated TG levels (*n* = 34), and 25.8% with elevated FBG (*n* = 33).

### Anthropometric characteristics

**Fig. 1:**
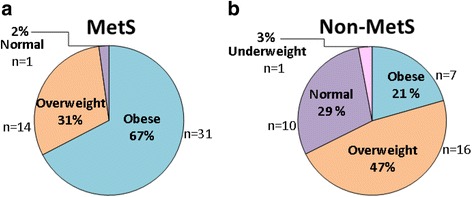
Distribution of women in (**a**) the MetS and (**b**) the non-MetS groups according to the respective BMI categories. MetS-metabolic syndrome, non-MetS-non-metabolic syndrome

In the subsample (*n* = 80), women in the MetS group were significantly older (40.1 (8.9–13.5) vs 32.2 (6.4–10.4) years, *p* = 0.00056), and also displayed a significantly higher BM, BMI, WC, WHR, and fat mass (%) (*p* < 0.001 for all, except WHR: *p* = 0.02), compared to the non-MetS counterparts (Table 1). With reference to BMI (Fig. 1), approximately two thirds of the MetS population were classified as obese (67.0%), and 31.0% as overweight, (Fig. 1a). In the non-MetS group, 47.0% of the females were overweight and 21.0% were obese (Fig. 1b).

**Table 1:** Summary of anthropometric and BIA characteristics for the MetS and non-MetS groups

Variable	MetS (*n* = 46)	Non-MetS (*n* = 34)	*p*-value
Age (years)	40.1 (8.9–13.5)	32.2 (6.4–10.4)	*p* = 0.00056
BM (kg)	84.81 (14.3–21.7)	69.10 (16.1–26.3)	*p* = 0.00033
BMI (kg/m^2^)	34.4 (5.2–7.9)	27.4 (5.8–9.4)	*p* = 0.000014
WC (cm)	93.10 (8.7–13.2)	79.48 (10.7–17.5)	*p* = 0.000002
WHR	0.81 (0.053–0.078)	0.78 (0.068–0.11)	*p* = 0.02
Fat mass (%)	42.42 (9.22–14.00)	32.04 (12.9–21.0)	*p* = 0.00097

All values are presented as mean and 95% Confidence Intervals (CI). Student t-tests were employed, and *p* < 0.05 was considered statistically significant. BM-Body mass, BMI-Body mass index, MetS-metabolic syndrome, Non-MetS-non-metabolic syndrome, ns = not significant, WC-waist circumference, WHR-waist-hip-ratio

### Biochemical blood parameters

In the subsample (*n* = 80), women with the MetS revealed significantly higher fasting insulin (*p* = 0.02), and FAI (*p* = 0.03), while SHBG were significantly lower compared to their non-MetS counterparts (*p* = 0.0083) (Table 2). All other parameters were found to be not significantly different (Table 2).

**Table 2:** Summary of physiological and biochemical blood parameters for the MetS and non-MetS groups

Variable	MetS ( *n* = 46)	Non-MetS ( *n* = 34)	*p*-value
Fasting Insulin (mIU/L)	35.69 (35.82–54.39)	17.36 (9.7–15.9)	*p* = 0.02
CRP (mg/L)^a^	10.86 (7.9–12.3)	6.68 (4.8–8.8)	*p* = 0.18
IGF-1 (ng / mL) ^b^	79.50 (56.16–88.57)	61.85 (44.6–82.8)	*p* = 0.41
SHBG (nmol/L)	42.57 (20.51–31.15)	73.84 (58.9–96.1)	*p* = 0.0083
DO	1.20 ± 0.80–7.75	0.70 ± 0.30–1.70	*p* = 0.03

All values are presented as mean and 95% Confidence Intervals (CI) with the exception of FAI, which is presented as median ± IQR. Student t-tests were employed for all parameters except for FAI, where Mann-Whitney U tests was employed. *p* < 0.05 was considered as statistically significant.^a^Only *n* = 64 samples included for analysis. ^b^Only *n* = 61 samples included for analysis. CRP-C-reactive protein, FAI-free androgen index, IGF-1-insulin-like growth factor-1, MetS-metabolic syndrome, non-MetS-non-metabolic syndrome, SHBG-sex hormone binding globulin

**Fig. 2:**
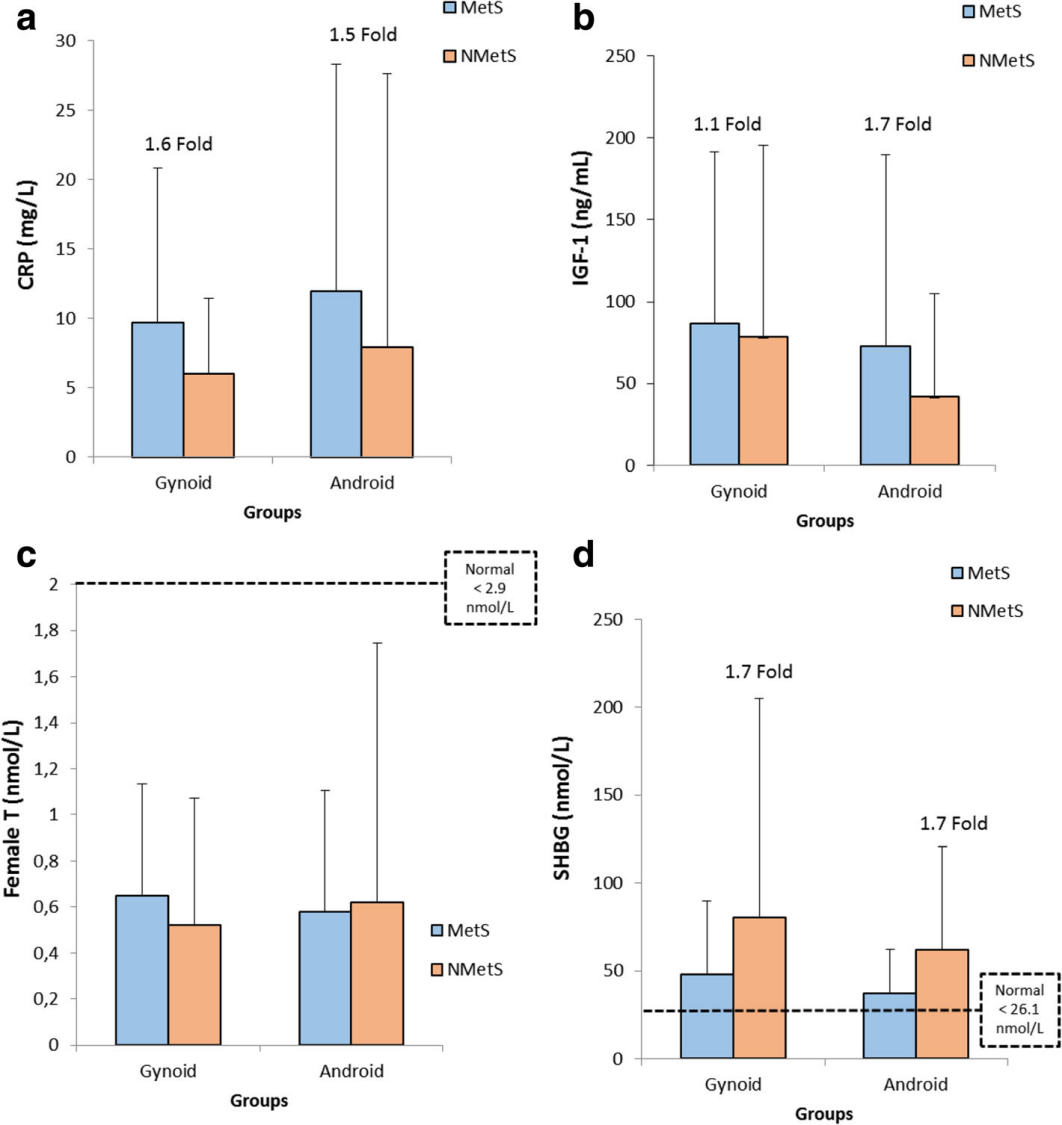
Blood analyses for (**a**) CRP, (**b**) IGF-1, (**c**) female Testosterone and (**d**) SHBG for the different body shape and metabolic status groups. The dotted lines represent normal cut-off criteria [37]. CRP-C-reactive protein, IGF-1-insulin-like growth factor-1, MetS-metabolic syndrome, Non-MetS-non-metabolic syndrome, SHBG-Sex hormone binding globulin, T-female testosterone

When considering the association of both metabolic status and body shape on various blood-specific parameters compared between the four respective groups, no significant differences were reported for CRP, IGF-1, female testosterone, or SHBG (Fig. 2a-d). However, even though no significance was observed, women with the MetS (irrespective of body shape) displayed at least a 1.5 fold higher CRP level (Fig. 2a), compared to their non-MetS group. Women in both the non-MetS groups displayed a 1.7 fold higher SHBG level compared to their MetS counterparts (Fig. 2d).

### Differences between respective groups according to both metabolic syndrome and body shape

For a more thorough investigation into the association of metabolic status and body shape, various anthropometric, BIA and blood-specific parameters were compared between the four respective groups (*n* = 80): MetSA, MetSG, NMetSA and NMetSG.

### Body composition

**Fig. 3:**
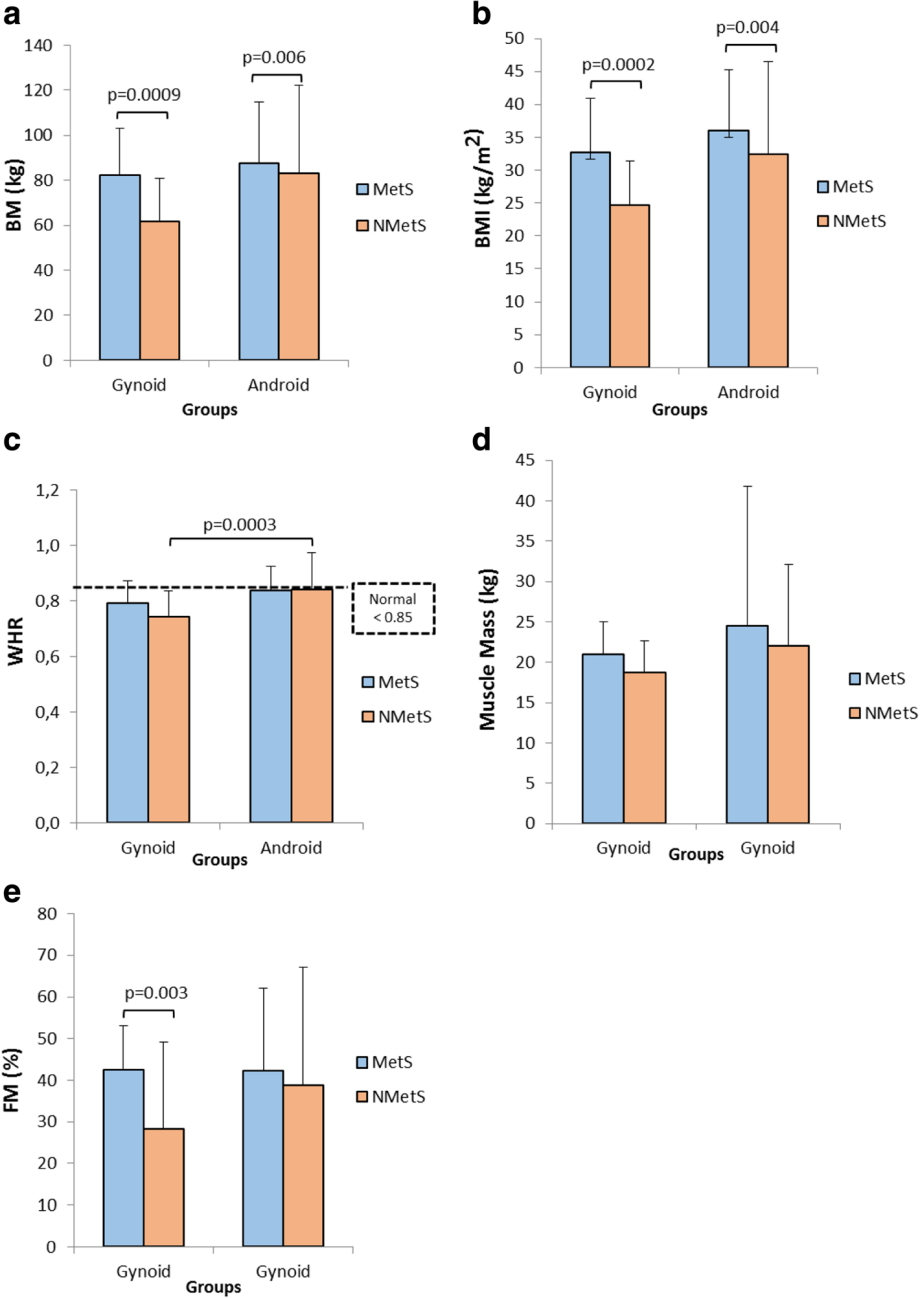
Body composition measurements for (**a**) BM, (**b**) BMI, (**c**) WHR, (**d**) Muscle mass, and (**e**) Fat mass (%). The dotted lines represent normal cut-off criteria [38]. BM-Body mass, BMI-Body mass index, MetS-metabolic syndrome, non-MetS-non-metabolic syndrome, WC-waist circumference, WHR-waist-hip-ratio

Women in both MetS groups (irrespective of body shape) (Fig. 3a and b) showed significantly higher BM and BMI compared to the non-MetS groups (*p* < 0.01), whereas women in the NMetSA group showed a significantly higher WHR compared to those from the NMetSG group (*p* = 0.0003) (Fig. 3c). A significantly higher fat mass (%) (Fig. 3e) was observed in the MetSG group compared to the NMetSG group (*p* = 0.003). No differences were observed for muscle mass between any groups (Fig. 3d).

### MetS risk factor measurements

**Fig. 4:**
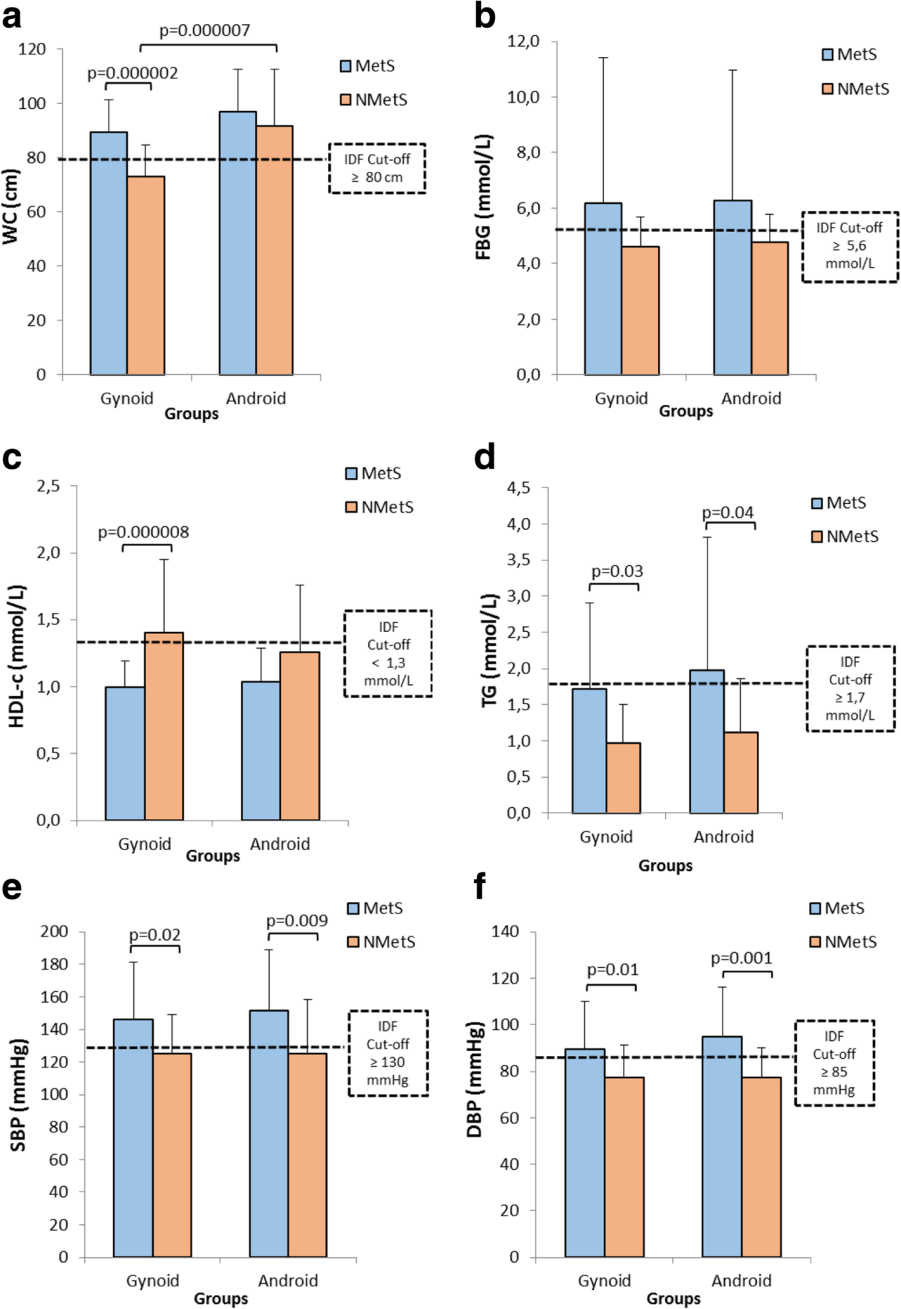
MetS risk factors according to IDF criteria for (**a**) WC, (**b**) FBG, (**c**) HDL-c, (**d**) TG, (**e**) SBP, and (**f**) DBP. The dotted lines represent IDF cut-off criteria (IDF, 2006). DBP-diastolic blood pressure, FBG-fasting blood glucose, HDL-c- high density lipoprotein-cholesterol, MetS-metabolic syndrome, non-MetS-non-metabolic syndrome, SBP-systolic blood pressure, TG- triglyceride, WC- waist circumference

Women in the MetSG group displayed a significantly larger WC compared to the NMetSG counterparts (*p* = 0.000002) (Fig. 4a), while the WC for the NMetSA group was significantly larger than that of the NMetSG group (*p* = 0.000007) (Fig. 4a). No significant differences were observed for FBG between any groups (Fig. 4b). The women in the MetSG group furthermore showed both a significantly lower HDL-c and elevated TG levels compared to the women in the NMetSG group (*p* = 0.000008 for HDL-c, and *p* = 0.03 for TG) (Fig. 4c and d). No other significant differences were observed for HDL-c, but TG levels were also significantly higher in the MetSA group vs the NMetSA group (*p* = 0.04) (Fig. 4c and d). The MetS groups displayed significant higher SBP and DBP vs the non-MetS groups, irrespective of body shape (Fig. 4e and f).

## Discussion

### Prevalence

More than 50% of the women in the current study (total population) were categorized with the MetS, which is considerably higher than what was previously reported by international [5, 8, 9], as well as other South African studies [6, 7]. Our results confirmed data reported in a local South African-based study [22], where it was shown that the prevalence (IDF criteria) of the MetS in a Capetonian urban black women population to be even higher (67.8%), however, the current population was specifically from farm working communities. The current study also reports that abdominal obesity (measured by WC) (68.8%), elevated blood pressure (66.4%) and decreased HDL-c (64.1%) were the most prevalent MetS risk factors, which is comparable to other South African studies [6, 7].

Studies have further proposed that abdominal obesity might be responsible; either independently, or through the induction of insulin resistance (via inflammatory mediators released from adipose tissue), to contribute to both the development of systemic hypertension and dyslipidaemia [6, 7, 16]. Since our population presented with an increased WC and overall higher prevalence of overweight, and obesity, this notion could also be plausible in this study. Obesity thus poses a major health problem in South African farm working women and may even predispose individuals to develop the MetS if it overlaps with other MetS risk factors. Since limited evidence exist with regards to the prevalence of the MetS, as well as its individual components in this study population as well as specific region, this study enabled us to partly describe and gain insight on the extent of the MetS in the Western Cape amongst women from farm working communities. It also allowed us to make substantial contributions regarding initiatives required to counter-act the rising prevalence of the MetS by increasing the awareness of the importance metabolic health in terms of health implications. Appropriate and cultural sensitive interventions are currently being developed to help address these public health issues.

Additionally, we determined if metabolic dysfunction and body fat distribution could predict differences in body composition, physiological and various blood (inflammatory, growth factor and steroid sex hormones) parameters to help identify the underlying pathophysiology, and/or associations.

### Body composition

We reported significantly higher BM, BMI, WC, WHR and fat mass (%) in the MetS groups compared to their non-MetS counterparts, which is well supported by others [23, 24]. During a chronic positive energy state, adipose tissue stores excess energy in the form of TG in adipocytes, this adipose tissue becomes dysfunctional (hyperplasia/hypertrophy) and eventually leads to an increase in fat mass [16]. A change in fat free mass may lead to changes in insulin sensitivity and glucose disposal, which contribute towards the development of the MetS and its components [25], which further exacerbate metabolic dysfunction.

### Physiological and blood parameters

Women in the MetS group showed significantly higher fasting insulin and FAI, while SHBG were significantly lower. Studies have suggested that the altered sex hormone profile may be ascribed to obesity and IR/hyperinsulinemia, which in turn can decrease oestrogen production and increase FAI by directly decreasing SHBG levels [17, 26, 27]. This might be a plausible explanation for our population, since the fasting insulin levels were also significantly higher in the MetS group.

### Metabolic syndrome showed associations on certain body compositional and MetS risk factor measurements in women with either gynoid or android body shapes

Evidence suggests that body shape, rather than total adiposity, potentially show strong clinical significance in the development of the MetS [10]. The android body shape is characterized by the presence of large abdominal fat deposits [12], whereas anthropometric indices (WC and WHR) increase as a result of an increase in VAT and subcutaneous adipose tissue [13]. These body composition changes, especially those associated with VAT, can therefore also increase the risk to for the MetS [10].

In the current study, the MetS showed a relationship on certain body compositional and MetS risk factors measurements in both body shape groups (android and gynoid). These discrepancies may be due to the following: firstly, our study had a relatively small sample size, which could have affected the statistical power, therefore did not reveal differences. Secondly, women in both the android and gynoid groups had relatively high BMIs, and we speculate that this could have accounted for the lack of differences in body compositional and blood parameters as seen in this study.

### IGF-1 axis: Metabolic syndrome and body shape combined does not predict differences in IGF-1

Adipose tissue distribution seems to play a role in the pathophysiology of the MetS through a correlation on growth factor levels [18]. Evidence on IGF-1 in relation to metabolic diseases is still controversial, i.e. low levels of IGF-1 have been suggested to have beneficial effects on glucose homeostasis and may also sensitise insulin actions, thereby decreasing metabolic disease risk [28]. Friedrich et al. (2013) showed that participants with the MetS had significantly higher IGF-1 levels vs healthy age-matched controls [18], whereas no significant differences were observed for IGF-1 between any of the respective groups in the current study, which is in agreement with the findings of Kabir et al. (2010) [29]. This suggests that although free IGF-1 may change due to adiposity, total IGF-1 remains within the reference range [30], and therefore remain unchanged. In addition, the observation of “no difference” includes the variability of IGF-1 levels found in this sample population. It should also be noted that IGF-1 levels are sensitive to age, gender, ethnic background, as well as degree of obesity [28].

### Inflammation: Metabolic syndrome and body shape does not predict differences in CRP

Adipose tissue distribution is also proposed to have a primary role in the pathophysiology of the MetS by affecting inflammatory mediators [32–34]. The MetS is characterized by a deregulated inflammatory profile, leading to a persistent low-grade inflammatory state [15]. An increase in pro-inflammatory markers in obesity [31], android body shape [32] and the MetS [15] have been well documented. One proposed mechanism states that an increase in adipose tissue, as a result of adipose tissue dysfunction and adipokine deregulation, can lead to an increase in interleukin-6 synthesis. This regulates hepatic CRP-synthesis and thereby increases CRP levels [33]. Seeing that these adipokines have such diverse functions, a dysregulation in the synthesis of these adipokines and their actions, in relation to obesity, have been linked to the MetS and its individual components [31, 32].

The current study contradicts this evidence, since we did not report any association between MetS, or body shape, on CRP levels, even though clear differences were observed for other body compositional measures of obesity. It is plausible that similar reasons could be at play for the no differences observed in IGF-1. Although no significance was observed, we did however observe the mean CRP levels in the MetS groups to be greater than 10 mg/L. This concentration is indicative of an underlying systemic infection and/or low-grade inflammation. Evidence shows that CRP levels greater than 10 mg/L in obesity have been documented and may be associated with overweight/obesity [34]. Thus, the increased CRP levels in the MetS group could be attributed to either being overweight/obese, an underlying systemic infection, or both; however, more research is needed to clarify these results.

### Sex hormone profile: Both metabolic syndrome and body shape does not predict variances in female T and SHBG

The MetS is characterized by an altered sex hormone profile, i.e. an increase in androgen concentration, increased female testosterone, FAI and decrease in SHBG [17, 26]. Androgen surplus has been associated with an increase in VAT and an android body shape, which increases the risk to develop the MetS [10, 17].

As a result of obesity, and its association with hyperinsulinemia, female testosterone can increase with a concomitant decrease in SHBG by having an effect on hepatic synthesis of SHBG [27]. However, no differences were observed for female testosterone, SHBG, as well as FAI. Since, hyperinsulinemia/hyperglycaemia has been shown to affect the sex hormone profile [35], the no differences observed for insulin and glucose; might explain the “no differences” observed for all the sex hormone parameters measured. Other confounding factors could include age, menopausal status, obesity, ovarian failure, polycystic ovarian syndrome, as well as the small sample size [36].

This current study provides significant contributions towards existing literature. To our knowledge, this is the first study to assess the prevalence of the MetS and its individual risk factors in a female farm working population in South Africa. It furthermore emphasized the problem of obesity in this gender specific South African farm working population, and the pathophysiology of the MetS in relation to adiposity and its distribution by including several factors (metabolic, growth and inflammatory factors, as well as sex hormone parameters), and measures of adiposity.

Although the current study has enabled us a better understanding of the MetS, the study was limited by the following: (i) the cross-sectional nature of the study hindered us to generalize our findings to the total population, or elucidate a causal relationship; (ii) CRP is a non-specific marker of inflammation; and (iii) the WC cut-off values used are not South-African specific. Lastly, we do acknowledge that each sub-group should at least consisted of *n* = 30 participants per group, to obtain a power of 80%, according to post-hoc sample size analysis. For future investigation we propose to include both pro- and anti-inflammatory markers, i.e. interleukin-6, interleukin-10 and TNF-alpha, combined with a full white blood cell count to rule out acute or chronic infections. We propose to include factors that could have confounded the sex hormone parameters including menstrual phase, age, menopausal status and parity.

## Conclusion

The prevalence of the MetS and its individual risk factors were found to be considerably high in this female farm worker population. Women with the MetS displayed a significantly exacerbated body composition and sex hormone profile. In addition, the MetS and body shape combined showed a relationship with certain body composition, physiological, as well as biochemical blood parameters, which in turn could exacerbate metabolic dysfunction. Although the effect of both metabolic status and body shape on inflammation, growth factors and sex hormone levels remains inconclusive, women need to recognize the burden of obesity and its associated metabolic dysfunction, and should be motivated to make changes regarding their metabolic health. Interventions should therefore be employed that are focused on metabolic health, which focusses specifically on exercise and nutrition in order to address the current status of the MetS in a culturally sensitive South African setting.

## Abbreviations

ANOVA: Analysis of variance
BEER: Bio-electrical impedance analysis
BM: Body mass
BMI: Body mass index
CANSA: Cancer Association of South Africa
CI: Confidence intervals
CRP: C-reactive protein
DBP: Diastolic blood pressure
ELISA: Enzyme-linked immunosorbent assay
DO: Free androgen index
FBG: Fasting blood glucose
HDL-c: High-density lipoprotein-cholesterol
IDF: International Diabetes Federation
IGF-1: Insulin-like growth factor-1
IQR: Interquartile range
IR: Insulin resistance
MetS: Metabolic syndrome
MetSA: Metabolic syndrome android group
MetSG: Metabolic syndrome gynoid group
NMetS: Non-metabolic syndrome group
NMetSA: Non-Metabolic syndrome android group
NMetSG: Non-Metabolic syndrome gynoid group
NRF: National Research Foundation
SBP: Systolic blood pressure
SEM: Standard error of the mean
SHBG: Sex hormone binding globulin
TG: Triglycerides
VAT: Visceral adipose tissue
vs: Versus
WC: Waist circumference
WHR: Waist-hip-ratio
